# Designing a response-over-continuous-intervention (ROCI) randomised trial: Implementation in the Phase 2C part (duration ranging) of the PARADIGM4TB trial

**DOI:** 10.1016/j.cct.2025.108002

**Published:** 2025-07-09

**Authors:** Tra My Pham, Angela M. Crook, Katie Rolfe, Patrick P.J. Phillips, Suzanne M. Dufault, Matteo Quartagno

**Affiliations:** a MRC Clinical Trials Unit at UCL, London, UK; b GSK, Stevenage, UK; c UCSF Center for Tuberculosis, University of California, San Francisco, San Francisco, CA, USA; d Division of Pulmonary and Critical Care Medicine, University of California, San Francisco, CA, USA; e Division of Biostatistics, University of California, San Francisco, San Francisco, CA, USA

**Keywords:** Randomised controlled trials, Response-over-continuous-intervention, ROCI, DURATIONS, Trial design, Tuberculosis

## Abstract

**Background/Aims:**

Treatments for tuberculosis (TB) are often long and complicated. Standard 2-arm non-inferiority trials have been used to evaluate shorter durations of treatment regimens. The new response-over-continuous-intervention (ROCI) trial design has recently been proposed as a practical alternative for optimising some continuous aspect (e.g. treatment duration) of treatment administration.

**Methods:**

We demonstrate the use of simulation for designing a ROCI trial in the TB setting. We use the Phase 2C part (duration ranging) of the PARADIGM4TB trial as a case study to illustrate the simulation procedure and the important design considerations to be explored in simulation. Phase 2C of PARADIGM4TB aims to optimise durations of novel treatment regimens, compared to a 6-month standard-of-care treatment regimen, with the aim to support advancement to Phase 3 trials.

**Results:**

A ROCI design randomising 200 patients to 5 equally spaced duration arms of the novel treatment regimen (with an additional 40 patients randomised to the standard-of-care treatment regimen) is sufficient to achieve reasonable power to identify the optimal duration in a range of scenarios. Modelling the duration-response curve with a fractional polynomial model of degree 1 improves power to select shorter durations compared with pairwise comparisons. A design with 5 durations of the novel regimen is preferred to a design with 3 durations, because of the improved operating characteristics in scenarios where the duration-response curve is not flat.

**Conclusions:**

The ROCI design is an appealing design option for TB treatment trials. The design of ROCI trials can be done by conducting simulation studies to explore key design considerations.

## 1. Introduction

Tuberculosis (TB) remains one of the global leading causes of death from an infectious disease, with 10.6 million new cases and 1.25 million deaths in 2023 [1]. Treatments for TB are often long (over many months) and onerous [2]. The long duration and severe side effects are associated with poor adherence in programmatic settings, which can lead to a higher risk of treatment failure or mycobacterial resistance [3]. Therefore, effort has been put into shortening the duration of treatment regimens for TB [4–6].

Traditionally, standard non-inferiority trials in which patients are randomised to 2 (or more) arms have been utilised in an attempt to find the optimal treatment durations. The non-inferiority design is motivated by the importance of maintaining the efficacy of treatment within an acceptable level, while benefiting from the reduced treatment administration. In this design, patients are randomised to receive either the standard (e.g. a 6-month regimen) or shortened duration/dose (e.g. a 4-month regimen) of either the same or a different treatment. A population-level summary measure is then calculated based on a specific outcome measured on these patients (e.g. difference in the risk of having an unfavourable outcome between two durations), which is then used to assess whether the shortened duration/dose is acceptable, i.e. if it is non-inferior to the standard duration/dose within a pre-specified non-inferiority margin. This approach poses 2 questions related to the trial's design and efficiency: (1) how do we pick the shortened duration/dose for comparison in a non-inferiority trial; and (2) should we treat the different durations/doses of the same treatment as independent?

The response-over-continuous-intervention (ROCI, formerly known as DURATIONS) trial design has recently been proposed as an alternative to the traditional 2-arm non-inferiority design [7,8]. This new design aims to address the above questions posed by the traditional approach, by: (1) randomising patients to multiple research arms within a reasonable range of treatment durations/doses; and (2) fitting a model to the multiple durations/doses (i.e. modelling a duration-response curve) to utilise information shared across them and thus increase efficiency.

The ROCI design is currently being used in trials across several therapeutic areas, such as to optimise the duration of oral antibiotic treatment for children hospitalised with severe pneumonia [9], or the frequency of immunotherapy administration for patients with advanced non-small cell lung cancer [10]. This paper discusses the implementation of the ROCI design in designing a trial with the aim to find the optimal treatment duration of the most promising novel regimens for TB to potentially bring forward to late-phase trials. The ROCI design as originally proposed aims to identify the shortest duration of a treatment that is non-inferior to the maximum duration of the same treatment [7,8] In this paper, we explore the inclusion of a control arm that could be different from the novel regimens being assessed.

The remainder of this paper is structured as follows: first, we introduce our motivating case study, the PARADIGM4TB trial; second, we present the steps involved in planning simulation studies to determine the sample size required for a ROCI trial; third, we describe the implementation of the ROCI design in designing Phase 2C of the PARADIGM4TB trial; finally, we conclude with a discussion and some potential extensions.

## 2. Motivating case study: PARADIGM4TB – Phase 2C (duration ranging)

**Fig. 1.**
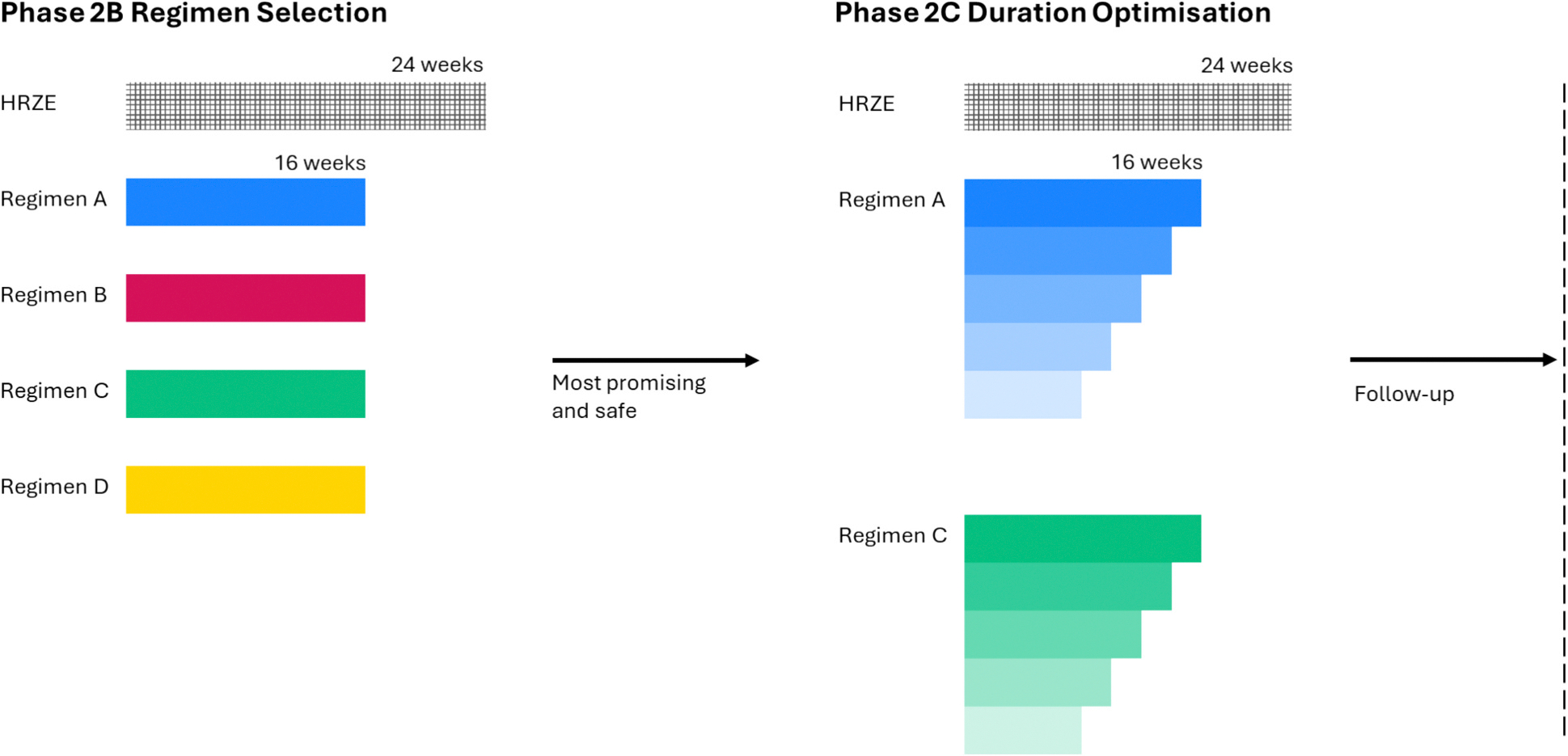
Schematic illustration of the design of the PARADIGM4TB trial (Phase 2B/C). Suppose that novel treatment regimens A and C with a duration of 16 weeks are brought forward after the regimen selection procedure in Phase 2B. In Phase 2C, different durations (8–16 weeks) of the selected regimens are compared against the standard-of-care HRZE regimen to support advancement to future Phase 3 trials.

The PARADIGM4TB trial is part of a programme of trials launched by UNITE4TB, a consortium for clinical drug and regimen development for TB [11]. PARADIGM4TB is a seamless Phase 2B/C trial which aims to identify novel treatment regimens with shortened durations compared with the standard-of-care HRZE regimen of 24 weeks (Fig. 1). The first part of the trial, Phase 2B (regimen selection), aims to identify 16-week regimens with the greatest potential based on assessment of quantitative sputum liquid culture and treatment failure/relapse [12].

Here we focus on Phase 2C (duration ranging) which aims to compare, among regimens selected for progression from Phase 2B, treatment durations of 8 to 16 weeks against the 24-week standard-of-care HRZE regimen, to support advancement to future Phase 3 trials. The primary efficacy endpoint is a binary composite endpoint of favourable/unfavourable status which is commonly used in TB treatment trials: the favourable status is defined as having culture negative status without failure, re-treatment or relapse at week 48 from randomisation; the unfavourable status is defined as either failure, re-treatment (not for re-infection), or relapse by week 48.

A ROCI trial design is therefore an appealing approach to determine the optimal durations for this part of the trial. We describe the process of designing the trial by answering the following questions:

1. What is a reasonable sample size for the trial?
2. Is modelling the duration-response curve necessary for improving the trial's operating characteristics?
3. If so, what is the preferred modelling strategy?
4. How many durations should patients be randomised to?

## 3. Design considerations to be explored in simulation

Quartagno et al. (2018) described the ROCI design in a setting where there is a standard-of-care treatment usually prescribed for a course of D_max_ (days/weeks), and it is believed that shorter durations of the same treatment might be acceptably as effective, with the shortest effective duration D_min_. A ROCI design aims to identify the shortest effective duration and assumes that the primary endpoint is a 0/1 binary indicator of cure. Patients are randomised to multiple duration arms, between and including D_max_ and D_min_, and the entire duration-response curve is modelled by fitting a pre-specified fractional polynomial logistic regression model in which the outcome is the binary indicator of cure and the only covariate is the duration, with up to 2 polynomial terms [13].

One way to design a ROCI trial is to use simulation to investigate the impact of various design choices. We follow the ADEMPI (Aims; Data generating mechanisms, Estimands, Methods of analysis, Performance measures, Implementation) framework for planning the simulation studies for this process [14].

### 3.1. Aims

The aim of Phase 2C of PARADIGM4TB is to optimise the durations of novel treatment regimens brought forward from Phase 2B. More specifically, the trial will assess whether any of these regimens can achieve similar efficacy to the standard-of-care HRZE regimen of 24 weeks, without being as long (N.B. the trial does not aim to shorten the HRZE regimen). This setting is slightly different from that previously considered in Quartagno et al. (2020) [8]; instead of comparing shorter durations to a control duration of the same treatment, here we are comparing several durations of a novel treatment regimen to a control treatment regimen.

The aim of these simulation studies is to identify the best design for the above task.

### 3.2. Data generating mechanisms

The design of the simulation studies requires specification of the following elements: control event risks, durations of novel treatment regimens and numbers of duration arms, upper limit of feasible sample sizes, shapes of the duration-response curve, and non-inferiority margins (Table 1). We simulate data according to data generating mechanisms that reflect as much as possible realistic and relevant scenarios.

#### 3.2.1. Control event risks

The primary outcome in Phase 2C of PARADIGM4TB is a binary composite endpoint of favourable/unfavourable status (see section 2). We consider two control event risks (i.e. the expected event risk in the standard-of-care HRZE regimen), 5 % and 10 %. The 5 % event risk is comparable with a recent drug-sensitive TB treatment trial where the same control was used [15]. However, since power would decrease for larger control event risks, for conservativeness, although unlikely, we consider the possibility of risk up to 10 %. Hence, outcome data are generated from a Bernoulli distribution with either 5 % or 10 % event probability.

#### 3.2.2. Durations of novel treatment regimens and numbers of duration arms

The duration of the standard-of-care HRZE regimen is 24 weeks, and novel regimens brought forward from Phase 2B to be compared with HRZE are of 16-week duration. We explore whether the duration of novel regimens could be feasibly reduced to as short as 8 weeks. Hence, we consider 2 scenarios: for each novel treatment regimen patients are randomised to either 5 equally spaced arms of 8, 10, 12, 14, and 16 weeks; or 3 equally spaced arms of 8, 12, and 16 weeks. The choice of 5 or 3 duration arms is also related to the methods of analysis which we will discuss later. Importantly, because of the methods used, results would apply to any scenario using the same number of equally spaced durations, irrespective of the precise durations.

#### 3.2.3. Sample sizes

We consider sample sizes of N_tot_dur_ = 200 and 300 patients to be randomised to the duration arms of each novel treatment regimen (split equally among the durations), with the same corresponding number of patients randomised to each duration being randomised to the standard-of-care HRZE regimen. These are thought to be feasible given the available resources of the trial. Therefore, if 5 durations of a novel treatment regimen are compared to HRZE and N_tot_dur_ = 200 patients are to be randomised to 5 duration arms, then there will be approximately N_arm_ = 40 patients in each arm, and the total sample size for the trial (5 duration arms and 1 control arm) will be N_overall_ = 240 patients. The same N_tot_dur_ = 200 patients to be randomised to 3 duration arms in a 3-duration design would lead to a larger total sample size of N_overall_ = 268 patients (3 duration arms and 1 control arm).

#### 3.2.4. Shapes of the duration-response curve

Similarly to standard non-inferiority trials, we want to ensure that the trial is well powered under the assumption that (1) each of the durations of the novel regimen is equally effective; and (2) the active event risk is equal to the control event risk. Therefore, we first assume a flat duration-response curve, and simulate outcomes for the duration arms from a Bernoulli distribution with probability of event equal to that in the control arm (i.e. 5 % or 10 %).

**Fig. 2.**
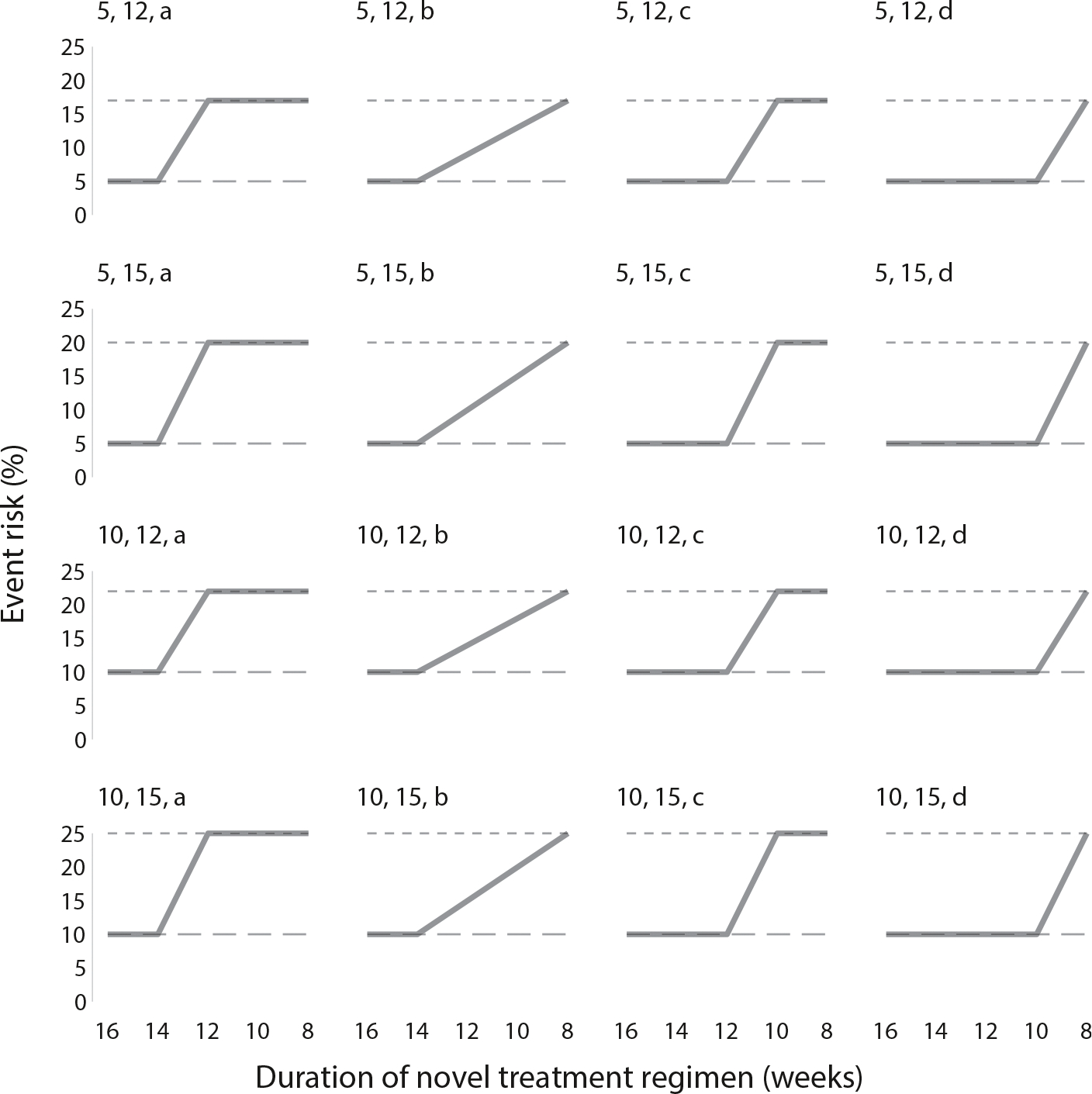
Shapes of the duration-response curve (a–d) for a design with 5 durations of the novel treatment regimen. Graph by control event risk (5 % or 10 %) and non-inferiority margin (12 % or 15 %); long dashed lines represent control event risks; short dashed lines are control event risks plus non-inferiority margins (e.g. 17 % for a control event risk of 5 % and a non-inferiority margin of 12 %). For example, the top left graph represents a scenario where the event rates are 5 % for the control, 16-week, and 14-week duration arms, and 17 % for the remaining (12-week, 10-week, 8-week) duration arms.

Additionally, we also consider scenarios where the duration-response curve is not flat (Fig. 2; Fig. S1), with shorter durations of the novel regimen leading to higher event risks than longer ones (e.g. 5 % event risk in the longest duration arm and 17 % event risk in the shortest ones).

#### 3.2.5. Non-inferiority margins

Margins of 10–12 % risk difference are generally acceptable to regulators for novel TB treatment regimens of shorter durations than the standard of care, but since this is a Phase-2 trial setting that evaluates novel treatment regimens, we consider slightly larger margins of either 12 % or 15 %, although we conjecture that a margin of 15 % might be considered too large.

### 3.3. Estimands

We are interested in determining the shortest treatment duration of a new regimen that is non-inferior to 6 months of treatment with control (using a pre-specified non-inferiority margin), as assessed by the proportion of patients with drug-sensitive TB experiencing an unfavourable outcome.

We assume that we would be interested in the treatment assignment only, i.e. we would use a treatment policy strategy for handling intercurrent events that are not already handled by a composite strategy in the primary outcome definition [16].

### 3.4. Methods of analysis

We compare 4 relevant approaches for estimating the risk difference between the control regimen and each duration of the novel regimen:

1. No model: we estimate a 90 % 2-sided Wald confidence interval (or the upper bound of the 1-sided 95 % confidence interval) around the difference in risk between the control arm and each duration arm.
2. Quadratic model: we fit a logistic regression model with a quadratic effect of duration on the log-odds of the outcome and use marginalisation to estimate the 2-sided 90 % confidence interval around the risk difference between each active duration and control using the model fit,

$$logit[p(y)]=\beta_{0}+\beta_{1}I[HRZE]+\beta_{2}D^{2},$$

where y is a binary variable representing the favourable/unfavourable outcome; I[HRZE] is a binary indicator taking values 1 if the patient is randomised to the control HRZE arm, and 0 otherwise; D is a continuous variable for the durations of the novel regimen (e.g. D takes value 8, 10, 12, 14, 16 in scenarios with 5 duration arms, and 0 when I[HRZE] takes value 1).

- Fractional polynomial of degree 1 (FP1): we use a flexible regression strategy known as fractional polynomial regression, which selects the best possible model out of a pool of powers. We then use marginalisation to estimate the 2-sided 90 % confidence interval around the risk difference using the model fit,

$$logit[p(y)]=\beta_{0}+\beta_{1}I[HRZE]+\beta_{2}D^{p_{1}},$$

where the power $p_{1}$ is chosen from the set { −2, −1, −0.5, 0, 0.5, 1, 2, 3}, with $p_{1}=0$ representing the logarithmic transformation, i.e. ln(D).

- Fractional polynomial of degree 2 (FP2): as above, but with 2 power terms, which is expected to lead to better fit but also more prone to overfitting with small sample sizes,

$$logit[p(y)]=\beta_{0}+\beta_{1}I[HRZE]+\beta_{2}D^{p_{1}}+\beta_{3}D^{p_{2}},$$

where the powers $p_{1}$ and $p_{2}$ are chosen from the set { −2, −1, −0.5, 0, 0.5, 1, 2, 3}.

Standard error calculations of the marginalisation procedure described above are based on the δ method, which gives a good approximation based on Taylor series expansions [8]. When using fractional polynomial modelling, the variability from selecting the powers is not reflected in the δ method. Bootstrap confidence interval can overcome this issue, where the fractional polynomial selection step is repeated in each bootstrapped sample to calculate the bootstrap standard errors [8]. However, bootstrap confidence interval is computationally more time consuming compared with the δ method. Therefore, we compare these approaches for calculating confidence intervals in a small selection of data generating mechanisms to explore whether their impact on the operating characteristics of the trial differs substantially (see Table S1).

Once all the risk differences are estimated with any of the above methods, since we expect the duration-response curve to be monotone, we adopt a closed testing approach, i.e. we first assess whether the 2-sided 90 % confidence interval for the risk difference for the longest duration vs control was within the non-inferiority margin and, only if it is, we then proceed with testing the second longest, and so on.

Note that fitting the FP1 and FP2 models above requires at least 4 and 6 degrees of freedom, respectively, hence we need to randomise patients to the standard-of-care HRZE regimen as well as either 3 or 5 durations of the novel regimen, respectively, to sufficiently estimate the parameters of these models. As a result, the FP2 model is not used when patients are randomised to only 3 durations of the novel regimen.

### 3.5. Performance measures

Designs and methods of analysis are compared based on “acceptable power”, i.e. the probability that a trial would find at least 1 duration of the novel regimen to be non-inferior to control within the pre-specified non-inferiority margin [8]. Further, we estimate both the mean and the median of the recommended durations from replications that found at least one acceptable duration. While the mean provides more information, the median would be important as well for scenarios where the distribution of recommended durations is expected to be skewed (e.g. the non-flat duration-response curves).

### 3.6. Implementation

For each data generating mechanism considered, we perform 1000 simulation repetitions. All simulations were run in Stata 17, except for simulations comparing bootstrap confidence intervals and the δ method which were run using Stata 15 provided by the UCL Myriad High Performance Computing Facility (Myriad@UCL). Stata code and simulated datasets for 1 data generating mechanism (N_tot_dur_ = 200 patients randomised to 5 durations in a flat duration-response curve scenario, with a control event risk of 5 % and a non-inferiority margin of 12 %; first row of Table 2) are included in the Supplementary Materials for illustration.

## 4. Results

Here we report the results of the simulations to answer our design questions stated above for Phase 2C of PARADIGM4TB. Using the δ method to calculate confidence intervals after marginalisation yields powers that are broadly comparable to the bootstrap confidence interval approach in the small selection of data generating mechanisms explored (Table S1). Therefore, for all of the following results, confidence intervals are calculated using the δ method.

### 4.1. What is a reasonable sample size for the trial?

**Fig. 3.**
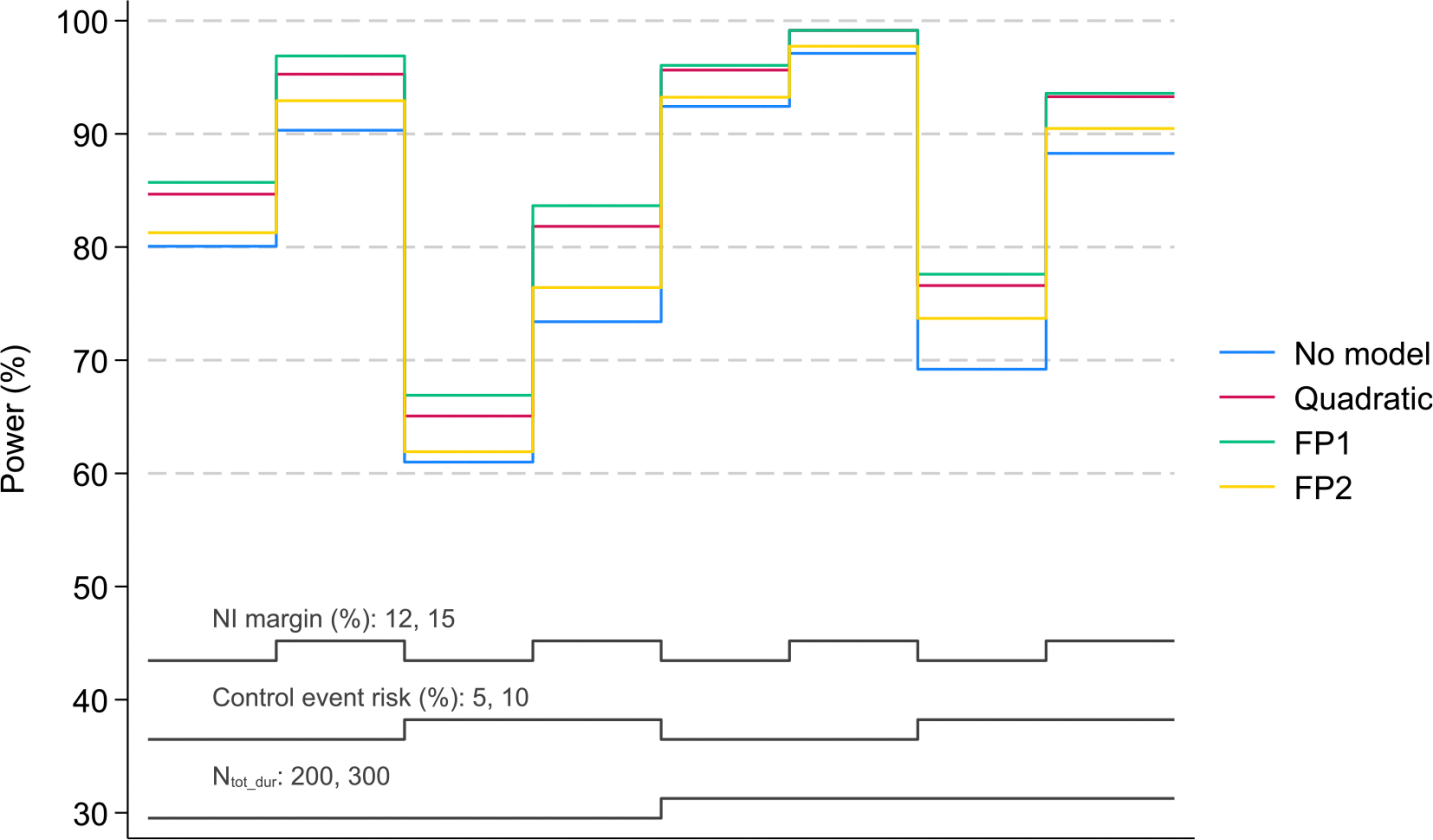
Power for a design with N_tot_dur_ = 200 or 300 patients randomised to 5 duration arms in a flat duration-response curve scenario (with the same number of patients in a given duration arm to be randomised to the standard-of-care HRZE regimen. Results are provided for different assumptions (HRZE control event risk and non-inferiority margins) and methods of analysis. Factors varied in simulations include N_tot_dur_ (200,300); HRZE control event risk (5 %, 10 %); and non-inferiority margin (12 %, 15 %); these are represented by the 3 grey lines at the bottom of the graph.

Table 2 and Fig. 3 show the results of the simulation studies for 5 equally spaced durations and the flat duration-response curve scenario, with sample sizes of N_tot_dur_ = 200 and N_tot_dur_ = 300 patients under different assumptions and using different methods of analysis. N_tot_dur_ = 200 patients provide reasonable (i.e. >80 %) levels of power irrespective of the modelling strategy. The only exceptions are when the control risk is as high as 10 % and the non-inferiority margin is set at 12 % (for all methods of analysis) or 15 % (for model-free comparison and FP2).

### 4.2. Is modelling the duration-response curve necessary for improving the trial's operating characteristics?

As seen in Table 2 and Fig. 3, power is always higher when using models to estimate the confidence intervals around the risk differences. In some cases the differences are substantial (i.e. up to 10 percentage points) across scenarios when using FP1 or a quadratic model.

### 4.3. If so, what is the preferred modelling strategy?

Power is higher when using FP1 (Table 2, Fig. 3). The decrease in power with FP2 suggests randomising N_tot_dur_ = 200 patients to 5 durations might not be enough to exploit the full potential of using a more complex model. While a quadratic model leads to similar power levels, it could lead to an increase in type 1 error if the assumed model turned out to be poorly specified.

### 4.4. How many durations should patients be randomised to?

**Fig. 4.**
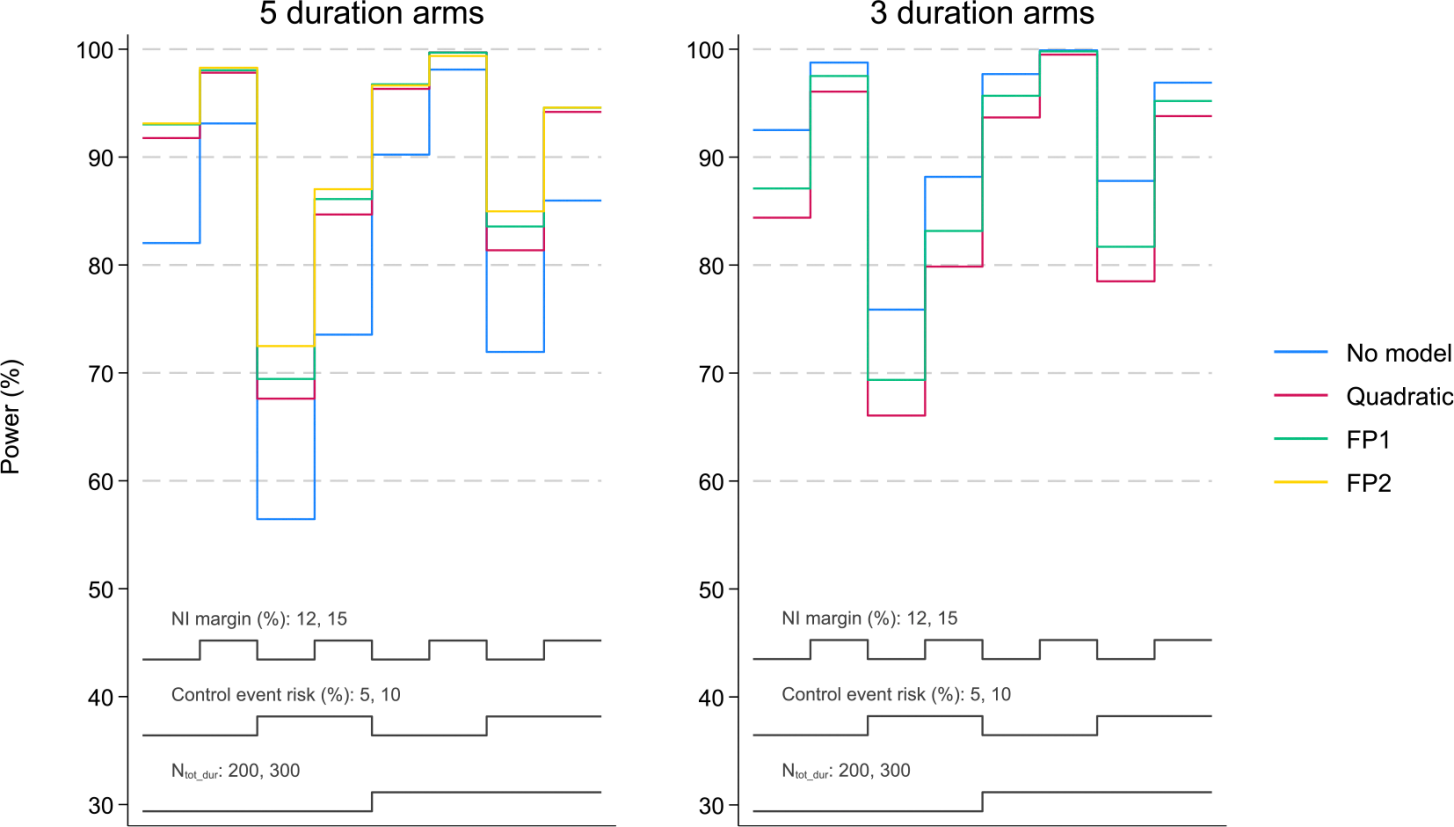
Power for a design with N_tot_dur_ = 200 or 300 patients randomised to 5 or 3 duration arms in a non-flat duration-response curve scenario (Figs. 2 and S1, shape a). Results are provided for different assumptions (HRZE control event risk and non-inferiority margins) and methods of analysis. Factors varied in simulations include N_tot_dur_ (200, 300); HRZE control event risk (5 %, 10 %); and non-inferiority margin (12 %, 15 %); these are represented by the 3 grey lines at the bottom of each graph panel.

Table 3 and Fig. 4 (left panel) show the results of simulation studies for a design comparing 5 durations of the novel regimen to the control under a non-flat simulation scenario, with event risk equal to 5 % (same as control event risk) in the 2 longest duration arms and 17 % (control event risk of 5 % + non-inferiority margin of 12 %) in the remaining duration arms. Table 4 and Fig. 4 (right panel) show the results under the same scenario for a design with 3 duration arms, where the same number of patients N_tot_dur_ is randomised to the durations, i.e. more patients are randomised to each arm. Results for other non-flat duration-response curves are presented in Tables S2, S4, S6 for 5 durations, and Tables S3 and S5 for 3 durations.

Power for the design with 5 durations using a FP1 model is generally at least comparable to power of the corresponding design with 3 durations using no model. Given the similar powers and despite the lower number of patients randomised to each duration with the 5-duration design, it is still preferred to a 3-duration design as it has greater scope for selecting an effective duration, i.e. the 14- and 10-week durations that are not available in the 3-duration design. These results generally hold in further simulation studies where, instead of fixing the total number of patients randomised to the duration arms (N_tot_dur_), we fix the overall number of patients in the trial (N_overall_), resulting in fewer patients being randomised to each arm in the 3-duration design and thus lower power (see Fig. S2 and Table S7 (parts a and e)). Also in further simulation studies, we compare power for the design with 5 durations to alternative designs with 4 durations (Table S7 (parts a–d), Fig. S3) and our conclusion remains.

## 5. Discussion

Using Phase 2C of the PARADIGM4TB trial as a case study, we have presented a strategy for designing trials with a ROCI design based on simulation, in order to optimise the duration of novel treatment regimens for TB. In the original ROCI design, the control arm is the maximum duration of the same treatment; in this paper we have proposed to implement the ROCI design in a TB treatment trial where the control arm is a separate regimen that is different from the novel treatment regimens whose durations are being assessed.

### 5.1. Summary of recommendations

Our simulation results have demonstrated that:

- A ROCI design randomising N_tot_dur_ = 200 patients to 5 equally spaced duration arms of the novel treatment regimen (with the same number of patients in a given duration arm to be randomised to the standard-of-care HRZE regimen) is sufficient to achieve reasonable power levels in a range of scenarios.
- Modelling the duration-response curve improves power compared to pairwise comparisons.
- A FP1 modelling strategy increases power to select lower durations.
- A design with 5 durations of the novel regimen is preferred to a design with 3 durations when the duration-response curve is not flat.

When designing trials with the aim to shorten the duration of novel treatment regimens for TB, a ROCI design is an appealing option and is more efficient than standard pairwise comparisons, since information shared across durations is utilised. Trial design and sample size determination may require performing simulation studies, so it is important to consider design parameters that are relevant to the setting of the trial, including the control event risks, durations of the novel treatment regimens, upper limit of feasible sample sizes, shape of the duration-response curve, and non-inferiority margins. Flexible modelling such as fractional polynomial regression generally improves power of the trial, however the choice of the powers used in the fractional polynomial model depends on the number of duration arms to which patients are randomised, e.g. ideally more than 5 duration arms should be used when the duration-response curve is modelled using FP2.

Within the context of TB treatment, while the duration with maximum efficacy would be of interest as well, shortening treatment duration may be more appealing to patients, e.g. a small improvement in efficacy could be less important than a large reduction in the length of treatment.

### 5.2. Considerations for implementation and potential extensions

We have presented a simulation-based approach for implementing the ROCI design; depending on the complexity of the design, performing large-scale simulation studies can potentially be complex and computationally intensive. As an alternative to this, we are working on developing analytical formulae for determining sample sizes for a ROCI design, which will make the implementation of the design more readily accessible.

In our simulation studies, we have explored scenarios in which there was (1) no difference between the control HRZE and the shortest duration arm; and (2) a relatively large difference of 12 % between the HRZE arm and shortest duration arm. While the design is well powered to detect durations equally effective to HRZE, which is our main aim, it is not powered to detect durations that lead to some loss of efficacy. An extension could be to explore the power of the design when the differences between durations are small, or there is a more gradual change with durations of increasing length.

The strategy for implementing the ROCI design presented in this paper is based on a frequentist framework that aims to identify the minimum duration of the novel treatment regimen that is non-inferior to the standard-of-care HRZE treatment. Future work could explore Bayesian methods that estimate the probabilities that each duration of the novel regimen is non-inferior to the control regimen within certain margins.

We note that there are simulation repetitions in which there are no events in the control arm; this is a limitation, particularly for smaller sample sizes. For implementation in Phase 2C of PARADIGM4TB, Bayesian analysis could be considered for handling this issue, given the use of prior distributions. Alternatively, for the frequentist analysis, an exact logistic regression method could be used.

Interim analyses could also be incorporated into a ROCI trial [10]. For Phase 2C of PARADIGM4TB, an interim analysis will be planned to assess futility, where duration arms with an unacceptably high proportion of unfavourable outcomes will be recommended for discontinuation, as will all shorter durations of the same regimen. Newly recruited patients will be randomised only to the remaining open durations. The impact of incorporating such an interim analysis on the overall power is expected to be small, but prior to implementation will be explored further in simulation. The long duration of follow-up required to assess the endpoint, together with anticipated recruitment times, and the number of arms for which recruitment is stopped, will need to be incorporated into the simulation design in order to adequately assess the operating characteristics of any proposed interim analysis.

Intercurrent events add extra complexities to the design and analysis of TB trials [17]. Potential intercurrent events and the choice of strategies for handling them should be discussed in the design stage of the trial, when defining the trial's estimand [3]. In TB trials, a composite strategy is often used to handle a number of intercurrent events such as treatment change/discontinuation for toxicity and TB-related death, where patients are classified as having an unfavourable outcome if they have the intercurrent events. The binary outcome used for implementing this ROCI design encompasses this unfavourable outcome which has incorporated the handling of some common intercurrent events. When planning a trial using simulation studies, the handling of intercurrent events should be considered and built into the simulation design.

## 6. Conclusions

The ROCI trial design is an appealing alternative to a standard 2-arm non-inferiority design for TB treatment trials, where the aim is to optimise the duration of novel or existing treatment regimens. Using Phase 2C of the PARADIGM4TB trial as a case study, we have demonstrated the use of simulation in implementing the ROCI design and discussed relevant design considerations.

## Supplementary Material

1

2

## Figures and Tables

**Table 1:** Design parameters used in simulation.

Design parameter	Level

Control (HRZE) event risks	5 % 10 %
Durations of novel treatment regimens	5 durations: 8, 10, 12, 14, 16 weeks 3 durations: 8, 12, 16 weeks
Sample sizes to be randomised to all durations of novel treatment regimens (as well as correspondong sample sizes per arm, and overall sample sizes for the trial)	N_tot_dur_ = 200 (e.g. for a 5-duration design with 1 control arm, N_arm_ = 40 and N_overall_ = 240), N_tot_dur_ = 300
Shapes of duration-response curve	Flat: Same event risk as the control arm for all duration arms (5 %, 10 %) Non-flat: same event risk as the control arm for longest duration arms, then event risk increases for shorter durations (Fig. 2, Fig. S1)
Non-inferiority margins	12 % 15 %

**Table 2:** Power and mean selected duration for a design with N_tot_dur_ = 200 or 300 patients randomised to 5 duration arms in a flat duration-response curve scenario. Results are provided for different assumptions (HRZE event risk and non-inferiority margins) and methods of analysis.

Sample size N_tot_dur_	HRZE control event risk	Non-inferiority margin	Power (%)				Mean selected durations in weeks				Number (%) of simulation repetitions with no events in HRZE arm	Number of simulation repetitions with model fitting error

	(%)	(%)	No model	Quadratic	FP1	FP2	No model	Quadratic	FP1	FP2	(excluded from power calculation)	(excluded from power calculation)
200 (40 per arm)	5	12	80.1	84.7	85.7	81.3	10.64	8.19	8.30	9.06	132 (13.2)	14 (1.4) [FP2]
	5	15	90.3	95.3	96.9	92.9	9.46	8.05	8.09	8.35	132 (13.2)	1 (0.1) [FP1], 19 (1.9) [FP2]
	10	12	61.0	65.1	66.9	61.9	12.56	9.06	9.13	10.50	18 (1.8)	0 (0)
	10	15	73.4	81.8	83.7	76.4	11.46	8.49	8.63	9.59	15 (1.5)	1(0.1) [FP2]
300 (60 per arm)	5	12	92.4	95.6	96.1	93.2	9.51	8.06	8.11	8.34	36 (3.6)	2 (0.2) [FP2]
	5	15	97.1	99.1	99.1	97.7	8.44	8.01	8.03	8.08	63 (6.3)	4 (0.4) [FP2]
	10	12	69.2	76.6	77.6	73.7	11.35	8.44	8.57	9.47	0 (0)	0 (0)
	10	15	88.3	93.3	93.6	90.5	10.10	8.15	8.21	8.58	2 (0.2)	0 (0)

Sample sizes presented refer to the number of patients randomised to the durations of the novel treatment regimen. The same number of patients will be randomised to the standard-of-care HRZE regimen, e.g. if N_tot_dur_ = 200 patients are randomised to 5 durations (N_arm_ = 40 in each arm), the total sample size of the trial is N_overall_ = 240 (5 duration arms and 1 control arm).

For results from fitting FP models: confidence intervals are calculated using the δ method.

Here we estimate a 90 % 2-sided Wald confidence interval (or the upper bound of the 1-sided 95 % confidence interval) around the difference in risk between the control arm and each duration arm.

**Table 3:** Power and median selected duration for a design with N_tot_dur_ = 200 or 300 patients randomised to 5 duration arms in a non-flat duration-response curve scenario (Fig. 2, first column, shape a). Results are provided for different assumptions (HRZE event risk and non-inferiority margins) and methods of analysis.

Sample size N_tot_dur_	HRZE control event risk	Non-inferiority margin	Power (%)				Median selected durations in weeks				Number (%) of simulation repetitions with no events in HRZE arm	Number of simulation repetitions with model fitting error

	(%)	(%)	No model	Quadratic	FP1	FP2	No model	Quadratic	FP1	FP2	(excluded from power calculation)	(excluded from power calculation)
200 (40 per am)	5	12	82.0	91.8	93.0	93.1	14	14	14	14	126 (12.6)	2 [FP2]
	5	15	93.1	97.8	98.1	98.3	14	12	14	14	128 (12.8)	2 [FP2]
	10	12	56.4	67.6	69.4	72.5	14	14	14	14	15 (1.5)	0 (0)
	10	15	73.5	84.7	86.1	87.0	14	14	14	14	21 (2.1)	0 (0)
300 (60 per arm)	5	12	90.2	96.3	96.7	96.6	14	14	14	14	48 (4.8)	0 (0)
	5	15	98.1	99.7	99.7	99.4	14	12	12	14	49 (4.9.)	0 (0)
	10	12	71.9	81.4	83.6	85.0	14	14	14	14	2 (0.2)	0 (0)
	10	15	86.0	94.2	94.6	94.6	14	14	14	14	2 (0.2)	0 (0)

Sample sizes presented refer to the number of patients randomised to the durations of the novel treatment regimen. The same number of patients will be randomised to the standard-of-care HRZE regimen, e.g. if N_tot_dur_ = 200 patients are randomised to 5 durations (N_arm_ = 40 in each arm), the total sample size of the trial is N_overall_ = 240 (5 duration arms and 1 control arm).

For results from fitting FP models: confidence intervals are calculated using the δ method.

Here we estimate a 90 % 2-sided Wald confidence interval (or the upper bound of the 1-sided 95 % confidence interval) around the difference in risk between the control arm and each duration arm.

**Table 4:** Power and median selected duration for a design with N_tot_dur_ = 200 or 300 patients randomised to 3 duration arms in a non-flat duration-response curve scenario (Fig. S1, first column, shape a). Results are provided for different assumptions (HRZE event risk and non-inferiority margins) and methods of analysis.

Sample size N_tot_dur_	HRZE control event risk	Non-inferiority margin	Power (%)			Median selected durations in weeks			Number (%) of simulation repetitions with no events in HRZE arm	Number (%) of simulation repetitions with model fitting error

	(%)	(%)	No model	Quadratic	FP1	No model	Quadratic	FP1	(excluded from power calculation)	(excluded from power calculation)
200 (67 per arm)	5	12	92.5	84.4	87.1	16	16	16	39 (0.39)	0 (0)
	5	15	98.8	96.1	97.5	16	16	16	34 (0.34)	0 (0)
	10	12	75.9	66.1	69.4	16	16	16	1 (0.1)	0 (0)
	10	15	88.2	79.9	83.2	16	16	16	2 (0.2)	0 (0)
300 (100 per arm)	5	12	97.7	93.7	95.7	16	16	16	4 (0.4)	0 (0)
	5	15	99.9	99.5	99.8	16	12	16	8 (0.8)	0 (0)
	10	12	87.8	78.5	81.7	16	16	16	0 (0)	0 (0)
	10	15	96.9	93.8	95.2	16	16	16	0 (0)	0 (0)

Sample sizes presented refer to the number of patients randomised to the durations of the novel treatment regimen. The same number of patients will be randomised to the standard-of-care HRZE regimen, e.g. if N_tot_dur_ = 300 patients are randomised to 3 durations (N_arm_ = 100 in each arm), the total sample size of the trial is N_overall_ = 400 (3 duration arms and 1 control arm).

For results from fitting FP models: confidence intervals are calculated using the δ method.

Here we estimate a 90 % 2-sided Wald confidence interval (or the upper bound of the 1-sided 95 % confidence interval) around the difference in risk between the control arm and each duration arm.

## Data Availability

Stata code and simulated datasets for 1 data generating mechanism are included in the Supplementary Materials for illustration.

## Appendix A. Supplementary data

Supplementary data to this article can be found online at https://doi.org/10.1016/j.cct.2025.108002.
