# Supplementary material for: Designing a response-over-continuous-intervention (ROCI) randomised trial: Implementation in the Phase 2C part (duration ranging) of the PARADIGM4TB trial
Source: Contemp Clin Trials. Author manuscript; Available in PMC 2026 Jun 3. (PMC13230843; doi:10.1016/j.cct.2025.108002)
Supplement: 1 [file NIHMS2169539-supplement-1.docx]

# Designing a response-over-continuous-interventions (ROCI) randomised trial: implementation in the Phase 2C part (duration ranging) of the PARADIGM4TB trial

## Supplementary materials

### Figures

**Figure S1**. Shapes of the duration-response curve (a–c) for a design with 3 durations of the novel treatment regimen.


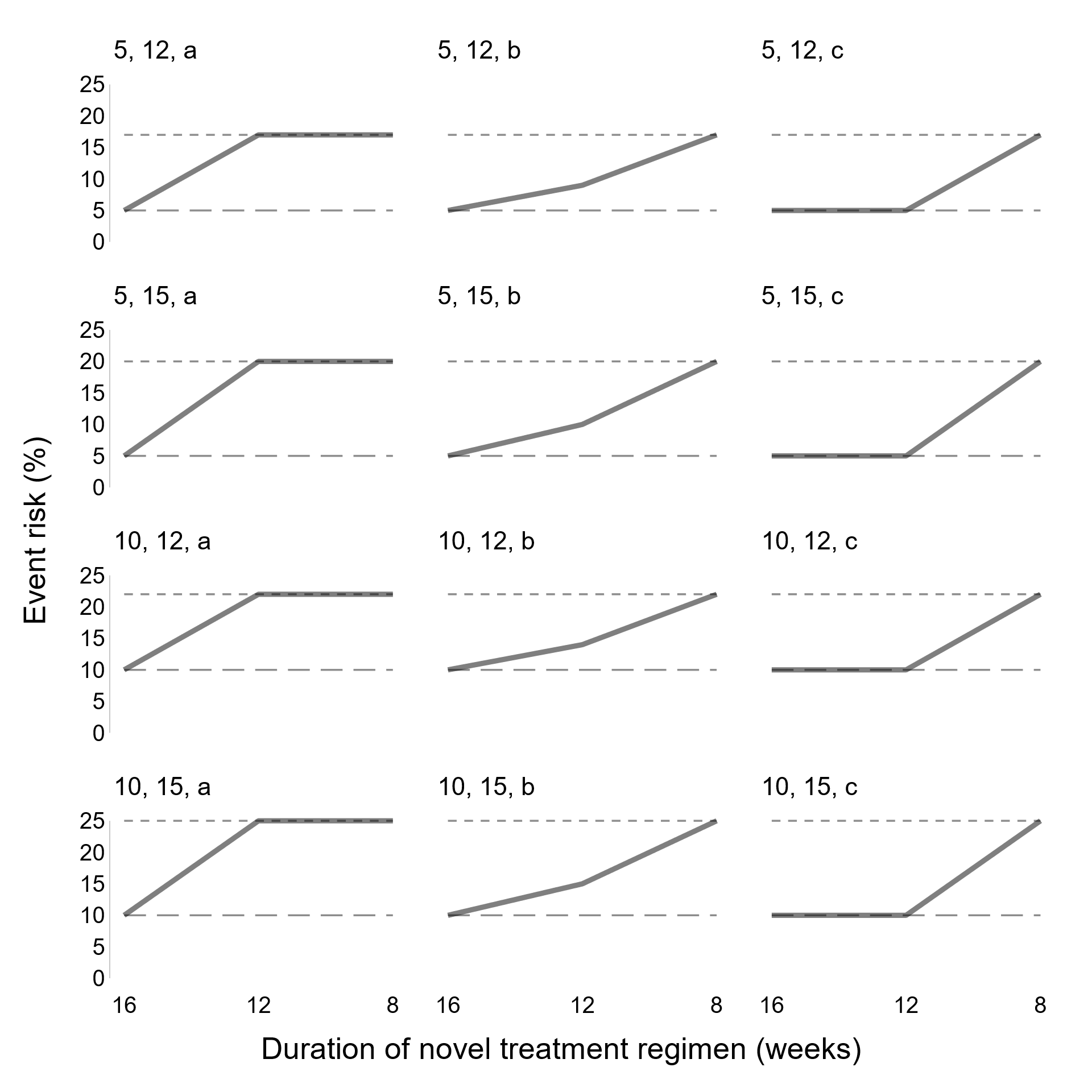


Graph by control event risk (5%, 10%) and non-inferiority margin (12%, 15%); long dashed lines represent control event risks; short dashed lines are control event risks plus non-inferiority margins (e.g. 17% for control event risk of 5% and non-inferiority margin of 12%). For example, the top left graph represents a scenario where the event rates are 5% for the control and the 16-week duration arms, and 17% for the remaining (12-week, 8-week) duration arms.

**Figure S2.** Power and median selection duration for a design with N_overall_ =240 or 360 patients randomised to 5 (a) or 3 (e) duration arms in a non-flat duration response curve scenario (Table S7). Results are provided for different assumptions (HRZE event risk (5%, 10%) and non-inferiority margins (12%, 15%); plotted at the bottom of each panel) and methods of analysis.

**Figure S3.** Power and median selection duration for a design with N_overall_ =240 or 360 patients randomised to 5 (a) or 4 (b–d) duration arms in a non-flat duration response curve scenario (Table S7). Results are provided for different assumptions (HRZE event risk (5%, 10%) and non-inferiority margins (12, 15%); plotted at the bottom of each panel) and methods of analysis.

### Tables

**Table S1**. Power and mean selected duration for a design with N_tot_dur_=200 or 300 patients randomised to 5 duration arms in a flat duration-response curve scenario. Results are provided for different assumptions (HRZE event risk and non-inferiority margins) and methods of analysis (with different approaches for obtaining the confidence intervals for FP1).

| Sample size N_tot_dur_ | HRZE control event risk | Non-inferiority margin | Power (%) | | | | | | Mean selected durations (weeks) | | | | | | | Number (%) of simulation repetitions with no events in HRZE arm | | Number (%) of simulation repetitions with model fitting error | |
| --- | --- | --- | --- | --- | --- | --- | --- | --- | --- | --- | --- | --- | --- | --- | --- | --- | --- | --- | --- |
|  | (%) | (%) | No model | Quad | FP1  (i) | FP1  (ii) | FP1  (iii) | FP2 | No model | Quad | FP1  (i) | FP1  (ii) | FP1  (iii) | FP2 | (excluded from power calculation) | | (excluded from power calculation) | |  |
| 200 (40 per arm) | 5 | 12 | 71.3 | 77.3 | 78.6 | 79.1 | 71.8 | 73.3 | 11.80 | 8.38 | 8.56 | 8.54 | 8.65 | 9.75 | 132 (13.2) | | 14 (1.4) [FP2] | |  |
|  | 5 | 15 | 85.5 | 0.91.9 | 92.5 |  |  | 87.0 | 10.11 | 8.11 | 8.18 |  |  | 8.63 | 132 (13.2) | | 1 (0.1) [FP1], 19 (1.9) [FP2] | |  |
|  | 10 | 12 | 46.7 | 52.9 | 55.1 |  |  | 50.0 | 13.51 | 9.67 | 9.76 |  |  | 11.42 | 18 (1.8) | | 0 (0) | |  |
|  | 10 | 15 | 64.1 | 72.3 | 74.2 |  |  | 66.2 | 12.43 | 8.85 | 9.08 |  |  | 10.35 | 15 (1.5) | | 1 (0.1) [FP2] | |  |
| 300 (60 per arm) | 5 | 12 | 86.4 | 91.4 | 92.7 |  |  | 88.6 | 10.31 | 8.12 | 8.21 |  |  | 8.65 | 36 (3.6) | | 2 (0.2) [FP2] | |  |
|  | 5 | 15 | 95.1 | 98.2 | 97.5 |  |  | 96.0 | 8.86 | 8.03 | 8.05 |  |  | 8.18 | 63 (6.3) | | 4 (0.4) [FP2] | |  |
|  | 10 | 12 | 59.8 | 65.9 | 69.2 |  |  | 62.2 | 12.23 | 8.76 | 8.88 |  |  | 10.14 | 0 (0) | | 0 (0) | |  |
|  | 10 | 15 | 79.9 | 87.7 | 89.2 | 88.0 | 87.7 | 83.0 | 11.06 | 8.27 | 8.35 | 8.38 | 8.36 | 8.97 | 2 (0.2) | | 0 (0) | |  |

- Sample sizes presented refer to the number of patients randomised to the durations of the novel treatment regimen. The same number of patients will be randomised to the standard-of-care HRZE regimen, e.g. if N_tot_dur_=200 patients are randomised to 5 durations (N_arm_=40 in each arm), the total sample size of the trial is N_overall_=240 (5 duration arms and 1 control arm).
- FP1: confidence intervals are calculated using (i) the δ method; (ii) normal bootstrap; (iii) bias-corrected bootstrap. FP2: confidence intervals are calculated using the δ method.
- We perform 960 bootstrap repetitions for each of the 1000 simulation repetitions for all 4 data generating mechanisms considered with sample sizes of N_tot_dur_=200 and N_tot_dur_ =300, HRZE control event risk of 5%, non-inferiority margins of 12% and 15%. The number of bootstrap repetitions is arbitrarily set to 4 times the total sample size of the trial of N_overall_=240).
- Number of bootstrap repetitions that fail (out of 960 bootstrap repetitions for each of the 1000 simulation repetitions):
  For first scenario (N_tot_dur_=200, HRZE=5%, NI margin=12%): range [0,455]; mean 169.5, percentiles 10%–21; 25%–49.5; 50%–129; 75%–340.5; 90%–363; For second scenario (N_tot_dur_=300, HRZE=10%, NI margin=15%): range [0,369]; mean 18.6, percentiles 10%–0; 25%–0; 50%–3; 75%–14; 90%–46.
- Here we estimate a 95% 2-sided Wald confidence interval (or the upper bound of the 1-sided 97.5% confidence interval) around the difference in risk between the control arm and each duration arm.

**Table S2**. Power and median selected duration for a design with N_tot_dur_=200 or 300 patients randomised to 5 duration arms in a non-flat duration-response curve scenario (Figure 2, second column, shape b). Results are provided for different assumptions (HRZE event risk and non-inferiority margins) and methods of analysis.

| Sample size N_tot_dur_ | HRZE control event risk | Non-inferiority margin | Power  (%) | | | | Median selected durations  (weeks) | | | | Number (%) of simulation repetitions with no events in HRZE arm | Number (%) of simulation repetitions with model fitting error |
| --- | --- | --- | --- | --- | --- | --- | --- | --- | --- | --- | --- | --- |
|  | (%) | (%) | No model | Quadratic | FP1 | FP2 | No model | Quadratic | FP1 | FP2 | (excluded from power calculation) | (excluded from power calculation) |
| 200 (40 per arm) | 5 | 12 | 81.3 | 96.3 | 96.3 | 87.2 | 14 | 12 | 12 | 12 | 130 (13.0) | 0 (0) |
|  | 5 | 15 | 92.3 | 99.0 | 99.1 | 95.2 | 12 | 12 | 12 | 12 | 117 (11.7) | 0 (0) |
|  | 10 | 12 | 58.7 | 79.5 | 79.7 | 63.8 | 14 | 14 | 12 | 14 | 20 (2.0) | 0 (0) |
|  | 10 | 15 | 75.2 | 91.9 | 92.5 | 80.5 | 14 | 12 | 12 | 12 | 16 (1.6) | 0 (0) |
| 300 (60 per arm) | 5 | 12 | 90.9 | 98.9 | 99.1 | 93.9 | 12 | 12 | 12 | 12 | 49 (4.9) | 0 (0) |
|  | 5 | 15 | 97.5 | 00.0 | 100.0 | 98.8 | 12 | 12 | 10 | 12 | 57 (5.7) | 0 (0) |
|  | 10 | 12 | 70.5 | 89.6 | 90.1 | 76.3 | 14 | 12 | 12 | 12 | 4 (0.4) | 0 (0) |
|  | 10 | 15 | 86.3 | 98.3 | 97.9 | 90.7 | 12 | 12 | 12 | 12 | 2 (0.2) | 0 (0) |

- Sample sizes presented refer to the number of patients randomised to the durations of the novel treatment regimen. The same number of patients will be randomised to the standard-of-care HRZE regimen, e.g. if N_tot_dur_=200 patients are randomised to 5 durations (N_arm_=40 in each arm), the total sample size of the trial is N_overall_=240 (5 duration arms and 1 control arm).
- For results from fitting FP models: confidence intervals are calculated using the δ method.
- Here we estimate a 90% 2-sided Wald confidence interval (or the upper bound of the 1-sided 95% confidence interval) around the difference in risk between the control arm and each duration arm.

**Table S3**. Power and median selected duration for a design with N_tot_dur_=200 or 300 patients randomised to 3 duration arms in a non-flat duration-response curve scenario (Figure S1, second column, shape b). Results are provided for different assumptions (HRZE event risk and non-inferiority margins) and methods of analysis.

| Sample size  N_tot_dur_ | HRZE control event risk | Non-inferiority margin | Power  (%) | | | Median selected durations  (weeks) | | | Number (%) of simulation repetitions with no events in HRZE arm | Number (%) of simulation repetitions with model fitting error |
| --- | --- | --- | --- | --- | --- | --- | --- | --- | --- | --- |
|  | (%) | (%) | No  model | Quadratic | FP1 | No  model | Quadratic | FP1 | (excluded from power calculation) | (excluded from power calculation) |
| 200 (67 per arm) | 5 | 12 | 93.2 | 97.1 | 97.2 | 12 | 12 | 12 | 29 (2.9) | 0 (0) |
|  | 5 | 15 | 98.7 | 99.5 | 99.4 | 12 | 12 | 12 | 50 (5.0) | 0 (0) |
|  | 10 | 12 | 7.69 | 83.8 | 84.8 | 16 | 12 | 12 | 2 (0.2) | 0 (0) |
|  | 10 | 15 | 90.4 | 93.7 | 94.6 | 12 | 12 | 12 | 2 (0.2) | 0 (0) |
| 300 (100 per arm) | 5 | 12 | 98.6 | 99.4 | 99.4 | 12 | 12 | 12 | 14 (1.4) | 0 (0) |
|  | 5 | 15 | 99.7 | 99.9 | 99.9 | 12 | 12 | 12 | 5 (0.5) | 0 (0) |
|  | 10 | 12 | 86.9 | 92.6 | 93.6 | 12 | 12 | 12 | 0 (0) | 0 (0) |
|  | 10 | 15 | 97.8 | 98.9 | 99.1 | 12 | 12 | 12 | 0 (0) | 0 (0) |

- Sample sizes presented refer to the number of patients randomised to the durations of the novel treatment regimen. The same number of patients will be randomised to the standard-of-care HRZE regimen, e.g. if N_tot_dur_=300 patients are randomised to 3 durations (N_arm_=100 in each arm), the total sample size of the trial is N_overall_=400 (3 duration arms and 1 control arm).
- For results from fitting FP models: confidence intervals are calculated using the δ method.
- Here we estimate a 90% 2-sided Wald confidence interval (or the upper bound of the 1-sided 95% confidence interval) around the difference in risk between the control arm and each duration arm.

**Table S4**. Power and median selected duration for a design with N_tot_dur_=200 or 300 patients randomised to 5 duration arms in a non-flat duration-response curve scenario (Figure 2, third column, shape c). Results are provided for different assumptions (HRZE event risk and non-inferiority margins) and methods of analysis.

| Sample size N_tot_dur_ | HRZE control event risk | Non-inferiority margin | Power  (%) | | | | Median selected durations  (weeks) | | | | Number (%) of simulation repetitions with no events in HRZE arm | Number (%) of simulation repetitions with model fitting error |
| --- | --- | --- | --- | --- | --- | --- | --- | --- | --- | --- | --- | --- |
|  | (%) | (%) | No model | Quadratic | FP1 | FP2 | No model | Quadratic | FP1 | FP2 | (excluded from power calculation) | (excluded from power calculation) |
| 200 (40 per arm) | 5 | 12 | 83.7 | 98.4 | 99.0 | 88.8 | 12 | 12 | 12 | 12 | 139 (13.9) | 9 (0.9) [FP2] |
|  | 5 | 15 | 91.8 | 99.8 | 99.9 | 94.0 | 12 | 12 | 12 | 12 | 130 (13.0) | 14 (1.4) [FP2] |
|  | 10 | 12 | 57.5 | 81.7 | 82.4 | 64.9 | 14 | 14 | 12 | 14 | 18 (1.8) | 0 (0) |
|  | 10 | 15 | 72.2 | 94.5 | 95.1 | 78.2 | 12 | 12 | 12 | 12 | 20 (2.0) | 0 (0) |
| 300 (60 per arm) | 5 | 12 | 91.1 | 99.5 | 99.6 | 94.3 | 12 | 12 | 12 | 12 | 40 (4.0) | 1 (0.1) [FP2] |
|  | 5 | 15 | 97.8 | 100.0 | 100.0 | 99.3 | 12 | 12 | 10 | 12 | 47 (4.7) | 2 (0.2) [FP2] |
|  | 10 | 12 | 70.8 | 92.2 | 92.4 | 77.9 | 12 | 12 | 12 | 12 | 5 (0.5) | 0 (0) |
|  | 10 | 15 | 88.0 | 98.6 | 98.9 | 91.6 | 12 | 12 | 12 | 12 | 5 (0.5) | 0 (0) |

- Sample sizes presented refer to the number of patients randomised to the durations of the novel treatment regimen. The same number of patients will be randomised to the standard-of-care HRZE regimen, e.g. if N_tot_dur_=200 patients are randomised to 5 durations (N_arm_=40 in each arm), the total sample size of the trial is N_overall_=240 (5 duration arms and 1 control arm).
- For results from fitting FP models: confidence intervals are calculated using the δ method.
- Here we estimate a 90% 2-sided Wald confidence interval (or the upper bound of the 1-sided 95% confidence interval) around the difference in risk between the control arm and each duration arm.

**Table S5**. Power and median selected duration for a design with N_tot_dur_=200 or 300 patients randomised to 3 duration arms in a non-flat duration-response curve scenario (Figure S1, third column, shape c). Results are provided for different assumptions (HRZE event risk and non-inferiority margins) and methods of analysis.

| Sample size N_tot_dur_ | HRZE control event risk | Non-inferiority margin | Power  (%) | | | Median selected durations  (weeks) | | | Number (%) of simulation repetitions with no events in HRZE arm | Number (%) of simulation repetitions with model fitting error |
| --- | --- | --- | --- | --- | --- | --- | --- | --- | --- | --- |
|  | (%) | (%) | No  model | Quadratic | FP1 | No  model | Quadratic | FP1 | (excluded from power calculation) | (excluded from power calculation) |
| 200 (67 per arm) | 5 | 12 | 93.8 | 98.7 | 99.3 | 12 | 12 | 12 | 52 (5.2) | 0 (0) |
|  | 5 | 15 | 98.8 | 100.0 | 100.0 | 12 | 12 | 12 | 31 (3.1) | 0 (0) |
|  | 10 | 12 | 77.5 | 89.9 | 92.1 | 12 | 12 | 12 | 0 (0) | 0 (0) |
|  | 10 | 15 | 89.5 | 96.9 | 98.4 | 12 | 12 | 12 | 1 (0.1) | 0 (0) |
| 300 (100 per arm) | 5 | 12 | 98.3 | 99.8 | 99.9 | 12 | 12 | 12 | 6 (0.6) | 0 (0) |
|  | 5 | 15 | 99.6 | 100.0 | 100.0 | 12 | 12 | 12 | 9 (0.9) | 0 (0) |
|  | 10 | 12 | 88.0 | 96.2 | 97.1 | 12 | 12 | 12 | 1 (0.1) | 0 (0) |
|  | 10 | 15 | 97.1 | 99.7 | 99.7 | 12 | 12 | 12 | 0 (0) | 0 (0) |

- Sample sizes presented refer to the number of patients randomised to the durations of the novel treatment regimen. The same number of patients will be randomised to the standard-of-care HRZE regimen, e.g. if N_tot_dur_=300 patients are randomised to 3 durations (N_arm_=100 in each arm), the total sample size of the trial is N_overall_=400 (3 duration arms and 1 control arm).
- For results from fitting FP models: confidence intervals are calculated using the δ method.
- Here we estimate a 90% 2-sided Wald confidence interval (or the upper bound of the 1-sided 95% confidence interval) around the difference in risk between the control arm and each duration arm.

**Table S6**. Power and median selected duration for a design with N_tot_dur_=200 or 300 patients randomised to 5 duration arms in a non-flat duration-response curve scenario (Figure 2, column 4, shape d). Results are provided for different assumptions (HRZE event risk and non-inferiority margins) and methods of analysis.

| Sample size N_tot_dur_ | HRZE control event risk | Non-inferiority margin | Power  (%) | | | | Median selected durations  (weeks) | | | | | Number (%) of simulation repetitions with no events in HRZE arm | | Number (%) of simulation repetitions with model fitting error | | |
| --- | --- | --- | --- | --- | --- | --- | --- | --- | --- | --- | --- | --- | --- | --- | --- | --- |
|  | (%) | (%) | No model | Quadratic | FP1 | FP2 | | No model | Quadratic | FP1 | FP2 | | (excluded from power calculation) | | (excluded from power calculation) |  |
| 200 (40 per arm) | 5 | 12 | 81.1 | 97.7 | 98.2 | 79.8 | | 10 | 10 | 10 | 10 | | 147 (14.7) | | 3 (0.3) [FP1], 6 (0.6) [FP2] |  |
|  | 5 | 15 | 91.3 | 99.5 | 99.8 | 89.9 | | 10 | 10 | 10 | 10 | | 113 (11.3) | | 2 (0.2) [FP1], 4 (0.4) [FP2] |  |
|  | 10 | 12 | 57.8 | 79.4 | 84.6 | 52.7 | | 14 | 12 | 12 | 10 | | 20 (2.0) | | 0 (0) |  |
|  | 10 | 15 | 71.9 | 92.8 | 95.7 | 69.1 | | 12 | 10 | 10 | 10 | | 22 (2.2) | | 0 (0) |  |
| 300 (60 per arm) | 5 | 12 | 90.6 | 99.2 | 99.5 | 89.6 | | 10 | 10 | 10 | 10 | | 50 (5.0) | | 0 (0) |  |
|  | 5 | 15 | 98.0 | 100.0 | 100.0 | 97.8 | | 10 | 10 | 10 | 10 | | 53 (5.3) | | 1 (0.1) [FP2] |  |
|  | 10 | 12 | 73.4 | 91.1 | 92.6 | 71.1 | | 10 | 10 | 10 | 10 | | 0 (0) | | 0 (0) |  |
|  | 10 | 15 | 87.9 | 98.6 | 99.4 | 86.6 | | 10 | 10 | 10 | 10 | | 2 (0.2) | | 0 (0) |  |

- Sample sizes presented refer to the number of patients randomised to the durations of the novel treatment regimen. The same number of patients will be randomised to the standard-of-care HRZE regimen, e.g. if N_tot_dur_=200 patients are randomised to 5 durations (N_arm_=40 in each arm), the total sample size of the trial is N_overall_=240 (5 duration arms and 1 control arm).
- For results from fitting FP models: confidence intervals are calculated using the δ method.
- Here we estimate a 90% 2-sided Wald confidence interval (or the upper bound of the 1-sided 95% confidence interval) around the difference in risk between the control arm and each duration arm.

**Table S7**. Power and median selection duration for a design with N_overall_ =240 or 360 patients randomised to 5, 4, and 3 duration arms and a control arm in a non-flat duration response curve scenario. Results are provided for different assumptions (HRZE event risk and non-inferiority margins) and methods of analysis.

1. 5 duration arms (16 weeks, 14 weeks, 12 weeks, 10 weeks, 8 weeks) & 1 control (HRZE) arm; the duration event risk is equal to the control event risk for the 16-week and 14-week duration arms, and equal to the control event risk + the non-inferiority margin for the 12-week, 10-week, and 8-week duration arms (Figure 2, column 1, shape a); these results are the same as presented in Table 3.

| Sample size N_overall_ | HRZE control event risk | Non-inferiority margin | Power (%) | | | | | Median selected duration (weeks) | | | | | Number (%) of simulation repetitions with no events in HRZE arm | Number (%) of simulation repetitions with model fitting error |
| --- | --- | --- | --- | --- | --- | --- | --- | --- | --- | --- | --- | --- | --- | --- |
|  | (%) | (%) | No model (Wald) | No model (NE10) | Quadratic | FP1 | FP2 | No model (Wald) | No model (NE10) | Quadratic | FP1 | FP2 | (excluded from power calculation) | (excluded from power calculation) |
| 240 (40 per arm) | 5 | 12 | 82.0 | 73.5 | 91.8 | 93.0 | 93.1 | 14 | 14 | 14 | 14 | 14 | 126 (12.6) | 2 (0.2) [FP2] |
|  | 5 | 15 | 93.1 | 86.0 | 97.8 | 98.1 | 98.3 | 14 | 14 | 12 | 14 | 14 | 128 (12.8) | 2 (0.2) [FP2] |
|  | 10 | 12 | 56.4 | 51.5 | 67.6 | 69.4 | 72.5 | 14 | 14 | 14 | 14 | 14 | 15 (1.5) | 0 (0) |
|  | 10 | 15 | 73.5 | 68.2 | 84.7 | 86.1 | 87.0 | 14 | 14 | 14 | 14 | 14 | 21 (2.1) | 0 (0) |
| 360 (60 per arm) | 5 | 12 | 90.2 | 86.3 | 96.3 | 96.7 | 96.6 | 14 | 14 | 14 | 14 | 14 | 48 (4.8) | 0 (0) |
|  | 5 | 15 | 98.1 | 97.2 | 99.7 | 99.7 | 99.4 | 14 | 14 | 12 | 12 | 14 | 49 (4.8) | 0 (0) |
|  | 10 | 12 | 71.9 | 68.1 | 81.4 | 83.6 | 85.0 | 14 | 14 | 14 | 14 | 14 | 2 (0.2) | 0 (0) |
|  | 10 | 15 | 86.0 | 83.4 | 94.2 | 94.6 | 94.6 | 14 | 14 | 14 | 14 | 14 | 2 (0.2) | 0 (0) |

- The event risk is HRZE control event risk for the control and 16-week duration arms, and HRZE event risk + non-inferiority margin for the other duration arms. For example, results for the first row is for a scenario where the control event risk and the event risk in the 16-week and 14-week duration arms is 5%, and the event risk for the remaining 3 duration arms is 17%.
- Here we estimate a 90% 2-sided Wald confidence interval (or the upper bound of the 1-sided 95% confidence interval) around the difference in risk between the control arm and each duration arm, except for the “No model” method where we also estimate a 90% 2-sided score confidence interval using the Newcombe method 10 (NE10).^1,2^ Generally the standard errors from a score test tend to be smaller than the Wald counterparts, leading to lower powers and thus larger sample sizes.
- For results from fitting FP models: confidence intervals are calculated using the δ method.

1. 4 duration arms (16 weeks, 12 weeks, 10 weeks, 8 weeks) & 1 control (HRZE) arm; the duration event risk is equal to the control event risk for the 16-week duration arm, and equal to the control event risk + the non-inferiority margin for the 12-week, 10-week, and 8-week duration arms.

| Sample size  N_overall_ | HRZE control event risk | Non-inferiority margin | Power (%) | | | | | Median selected duration (weeks) | | | | | Number (%) of simulation repetitions with no events in HRZE arm | Number (%) of simulation repetitions with model fitting error |
| --- | --- | --- | --- | --- | --- | --- | --- | --- | --- | --- | --- | --- | --- | --- |
|  | (%) | (%) | No model (Wald) | No model (NE10) | Quadratic | FP1 | FP2 | No model (Wald) | No model (NE10) | Quadratic | FP1 | FP2 | (excluded from power calculation) | (excluded from power calculation) |
| 240 (48 per arm) | 5 | 12 | 85.8 | 79.4 | 77.3 | 81.2 | 86.4 | 16 | 16 | 16 | 16 | 16 | 96 (9.6) | 5 (0.5) [FP2] |
|  | 5 | 15 | 94.8 | 91.7 | 88.0 | 90.9 | 95.4 | 16 | 16 | 16 | 16 | 16 | 91 (9.1) | 2 (0.2) [FP2] |
|  | 10 | 12 | 66.4 | 60.8 | 55.4 | 58.0 | 66.9 | 16 | 16 | 16 | 16 | 16 | 5 (0.5) | 1 (0.1) [FP2] |
|  | 10 | 15 | 81.5 | 78.7 | 72.1 | 75.1 | 81.6 | 16 | 16 | 16 | 16 | 16 | 14 (1.4) | 0 (0) |
| 360 (72 per arm) | 5 | 12 | 95.2 | 92.7 | 90.3 | 92.1 | 95.1 | 16 | 16 | 16 | 16 | 16 | 28 (2.8) | 0 (0) |
|  | 5 | 15 | 98.6 | 98.1 | 96.7 | 97.6 | 98.7 | 16 | 16 | 12 | 16 | 16 | 29 (2.9) | 0 (0) |
|  | 10 | 12 | 79.5 | 76.6 | 69.4 | 72.9 | 79.4 | 16 | 16 | 16 | 16 | 16 | 1 (0.1) | 0 (0) |
|  | 10 | 15 | 90.8 | 88.3 | 81.0 | 84.1 | 89.8 | 16 | 16 | 16 | 16 | 16 | 0 (0) | 0 (0) |

- The event risk is HRZE control event risk for the control and 16-week duration arms, and HRZE event risk + non-inferiority margin for the other duration arms. For example, results for the first row is for a scenario where the control event risk and the event risk in the 16-week duration arm is 5%, and the event risk for the remaining arms is 17%.
- Here we estimate a 90% 2-sided Wald confidence interval (or the upper bound of the 1-sided 95% confidence interval) around the difference in risk between the control arm and each duration arm, except for the “No model” method where we also estimate a 90% 2-sided score confidence interval using the Newcombe method 10 (NE10).^1,2^ Generally the standard errors from a score test tend to be smaller than the Wald counterparts, leading to lower powers and thus larger sample sizes.
- For results from fitting FP models: confidence intervals are calculated using the δ method.

1. 4 duration arms (16 weeks, 14 weeks, 10 weeks 8 weeks) & 1 control (HRZE) arm; the duration event risk is equal to the control event risk for the 16-week and 14-week duration arms, and equal to the control event risk + the non-inferiority margin for the 10-week, and 8-week duration arms.

| Sample size  N_overall_ | HRZE control event risk | Non-inferiority margin | Power (%) | | | | | Median selected duration (weeks) | | | | | Number (%) of simulation repetitions with no events in HRZE arm | Number (%) of simulation repetitions with model fitting error |
| --- | --- | --- | --- | --- | --- | --- | --- | --- | --- | --- | --- | --- | --- | --- |
|  | (%) | (%) | No model (Wald) | No model (NE10) | Quadratic | FP1 | FP2 | No model (Wald) | No model (NE10) | Quadratic | FP1 | FP2 | (excluded from power calculation) | (excluded from power calculation) |
| 240 (48 per arm) | 5 | 12 | 86.0 | 78.5 | 97.6 | 98.0 | 92.6 | 14 | 14 | 14 | 14 | 14 | 88 (8.8) | 11 (1.1) [FP2] |
|  | 5 | 15 | 94.4 | 90.0 | 99.2 | 99.5 | 97.5 | 14 | 14 | 14 | 14 | 14 | 89 (8.9) | 3 (0.3) [FP2] |
|  | 10 | 12 | 62.5 | 58.6 | 80.5 | 80.4 | 73.6 | 14 | 14 | 14 | 14 | 14 | 6 (0.6) | 0 (0) |
|  | 10 | 15 | 78.8 | 75.2 | 93.9 | 94.3 | 87.3 | 14 | 14 | 14 | 14 | 14 | 9 (0.9) | 0 (0) |
| 360 (72 per arm) | 5 | 12 | 93.0 | 90.9 | 98.9 | 99.1 | 96.9 | 14 | 14 | 14 | 14 | 14 | 23 (2.3) | 0 (0) |
|  | 5 | 15 | 99.2 | 98.1 | 100 | 100 | 99.6 | 14 | 14 | 14 | 14 | 14 | 39 (3.9) | 0 (0) |
|  | 10 | 12 | 77.2 | 74.9 | 91.7 | 91.3 | 86.9 | 14 | 14 | 14 | 14 | 14 | 0 (0) | 0 (0) |
|  | 10 | 15 | 90.7 | 89.5 | 99.1 | 98.9 | 95.0 | 14 | 14 | 14 | 14 | 14 | 0 (0) | 0 (0) |

- The event risk is HRZE control event risk for the control and 16-week duration arms, and HRZE event risk + non-inferiority margin for the other duration arms. For example, results for the first row is for a scenario where the control event risk and the event risk in the 16-week and 14-week duration arms is 5%, and the event risk for the remaining 2 duration arms is 17%.
- Here we estimate a 90% 2-sided Wald confidence interval (or the upper bound of the 1-sided 95% confidence interval) around the difference in risk between the control arm and each duration arm, except for the “No model” method where we also estimate a 90% 2-sided score confidence interval using the Newcombe method 10 (NE10).^1,2^ Generally the standard errors from a score test tend to be smaller than the Wald counterparts, leading to lower powers and thus larger sample sizes.
- For results from fitting FP models: confidence intervals are calculated using the δ method.

1. 4 duration arms (16 weeks, 14 weeks, 12 weeks, 8 weeks) & 1 control (HRZE) arm; the duration event risk is equal to the control event risk for the 16-week and 14-week duration arms, and equal to the control event risk + the non-inferiority margin for the 12-week and 8-week duration arms.

| Sample size N_overall_ | HRZE control event risk | Non-inferiority margin | Power (%) | | | | | Median selected duration (weeks) | | | | | Number (%) of simulation repetitions with no events in HRZE arm | Number (%) of simulation repetitions with model fitting error |
| --- | --- | --- | --- | --- | --- | --- | --- | --- | --- | --- | --- | --- | --- | --- |
|  | (%) | (%) | No model (Wald) | No model (NE10) | Quadratic | FP1 | FP2 | No model (Wald) | No model (NE10) | Quadratic | FP1 | FP2 | (excluded from power calculation) | (excluded from power calculation) |
| 240 (48 per arm) | 5 | 12 | 87.8 | 80.4 | 95.5 | 96.3 | 94.9 | 14 | 14 | 14 | 14 | 14 | 97 (9.7) | 4 (0.4) [FP2] |
|  | 5 | 15 | 93.9 | 91.4 | 98.2 | 98.9 | 98.6 | 14 | 14 | 12 | 12 | 14 | 96 (9.6) | 3 (0.3) [FP2] |
|  | 10 | 12 | 62.9 | 57.6 | 73.5 | 75.2 | 76.2 | 14 | 14 | 14 | 14 | 14 | 6 (0.6) | 0 (0) |
|  | 10 | 15 | 79.8 | 76.4 | 89.6 | 90.4 | 91.6 | 14 | 14 | 14 | 14 | 14 | 9 (0.9) | 0 (0) |
| 360 (72 per arm) | 5 | 12 | 93.8 | 90.9 | 98.9 | 99.1 | 98.6 | 14 | 14 | 12 | 12 | 14 | 23 (2.3) | 0 (0) |
|  | 5 | 15 | 99.4 | 98.3 | 99.9 | 100 | 100 | 14 | 14 | 12 | 12 | 14 | 1 (0.1) | 0 (0) |
|  | 10 | 12 | 75.8 | 73.8 | 87.2 | 88.2 | 89.9 | 14 | 14 | 14 | 14 | 14 | 1 (0.1) | 0 (0) |
|  | 10 | 15 | 90.7 | 89.0 | 97.1 | 97.2 | 97.1 | 14 | 14 | 14 | 14 | 14 | 0 (0) | 0 (0) |

- The event risk is HRZE control event risk for the control and 16-week duration arms, and HRZE event risk + non-inferiority margin for the other duration arms. For example, results for the first row is for a scenario where the control event risk and the event risk in the 16-week and 14-week duration arms is 5%, and the event risk for the remaining 2 duration arms is 17%.
- Here we estimate a 90% 2-sided Wald confidence interval (or the upper bound of the 1-sided 95% confidence interval) around the difference in risk between the control arm and each duration arm, except for the “No model” method where we also estimate a 90% 2-sided score confidence interval using the Newcombe method 10 (NE10).^1,2^ Generally the standard errors from a score test tend to be smaller than the Wald counterparts, leading to lower powers and thus larger sample sizes.
- For results from fitting FP models: confidence intervals are calculated using the δ method.

1. 3 duration arms (16 weeks, 12 weeks, 8 weeks) & 1 control (HRZE) arm; the duration event risk is equal to the control event risk for the 16-week duration arm, and equal to the control event risk + the non-inferiority margin for the 12-week and 8-week duration arms (Figure S1, first column, shape a).

| Sample size  N_overall_ | HRZE control event risk | Non-inferiority margin | Power (%) | | | | Median selected duration (weeks) | | | | Number (%) of simulation repetitions with no events in HRZE arm | Number (%) of simulation repetitions with model fitting error |
| --- | --- | --- | --- | --- | --- | --- | --- | --- | --- | --- | --- | --- |
|  | (%) | (%) | No model (Wald) | No model (NE10) | Quadratic | FP1 | No model (Wald) | No model (NE10) | Quadratic | FP1 | (excluded from power calculation) | (excluded from power calculation) |
| 240 (60 per arm) | 5 | 12 | 91.7 | 86.6 | 82.3 | 85.2 | 16 | 16 | 16 | 16 | 53 (5.3) | 0 (0) |
|  | 5 | 15 | 98.2 | 96.0 | 92.8 | 94.2 | 16 | 16 | 16 | 16 | 51(5.1) | 0 (0) |
|  | 10 | 12 | 72.2 | 68.2 | 62.1 | 65.3 | 16 | 16 | 16 | 16 | 4 (0.4) | 0 (0) |
|  | 10 | 15 | 84.5 | 82.5 | 75.6 | 78.5 | 16 | 16 | 16 | 16 | 3 (0.3) | 0 (0) |
| 360 (90 per arm) | 5 | 12 | 98.1 | 96.8 | 93.3 | 94.9 | 16 | 16 | 16 | 16 | 20 (2.0) | 0 (0) |
|  | 5 | 15 | 99.8 | 99.3 | 98.1 | 98.7 | 16 | 16 | 12 | 16 | 11 (1.1) | 0 (0) |
|  | 10 | 12 | 85.5 | 83.1 | 76.6 | 80.2 | 16 | 16 | 16 | 16 | 1 (0.1) | 0 (0) |
|  | 10 | 15 | 95.2 | 94.3 | 89.4 | 92.0 | 16 | 16 | 16 | 16 | 0 (0) | 0 (0) |

- The event risk is HRZE control event risk for the control and 16-week duration arms, and HRZE event risk + non-inferiority margin for the other duration arms. For example, results for the first row is for a scenario where the control event risk and the event risk in the 16-week duration arms is 5%, and the event risk for the remaining 2 duration arms is 17%.
- Here we estimate a 90% 2-sided Wald confidence interval (or the upper bound of the 1-sided 95% confidence interval) around the difference in risk between the control arm and each duration arm, except for the “No model” method where we also estimate a 90% 2-sided score confidence interval using the Newcombe method 10 (NE10).^1,2^ Generally the standard errors from a score test tend to be smaller than the Wald counterparts, leading to lower powers and thus larger sample sizes.
- For results from fitting FP models: confidence intervals are calculated using the δ method.

## References

1. Newcombe RG. Interval estimation for the difference between independent proportions: Comparison of eleven methods. *Stat Med* 1998; 17: 873--890.

2. Coveney J. RDCI: Stata module to calculate risk difference confidence intervals.
